# Effect of noise on the performance of arthroscopic simulator

**DOI:** 10.1016/j.sopen.2024.06.006

**Published:** 2024-07-05

**Authors:** Alexandre Czerwiec, Margot Vannier, Olivier Courage

**Affiliations:** a Orthopedic Department, Centre Hospitalier Universitaire de Rouen, 37 boulevard Gambetta, 76000 Rouen, France; b Biostatistics Department, Centre Hospitalier Universitaire de Rouen, 37 Boulevard Gambetta, 76000 Rouen, France; c Orthopedic Department, Hôpital privé de l'estuaire, 505 rue Irène Joliot Curie, 76620 Le Havre, France

**Keywords:** Arthroscopic simulator, Noise, Virtamed

## Abstract

**Background:**

Noise is omnipresent in the operating room. The average noise in the operating room generally ranges between 60 and 65 dB and can sometimes exceed 100 dB, despite the ARS (Agence Régionale de Santé) and WHO (World Health Organization) recommending levels of 35 dB(A). This study aimed to evaluate the effect of different kinds of background auditory stimuli on the performance of surgeons during an arthroscopic simulation task.

**Methods:**

Forty-seven surgeons with varying experience in arthroscopic surgery undertook different exercises under four different conditions: quiet, classical music, hard rock, and sustained chatter. All background auditory stimuli were set at 65 dB(A). Each participant underwent double randomization for the four sound stimuli and the four exercises to be performed. A musical questionnaire was also completed by each participant. Data related to each exercise included operating time in seconds, distance from the camera or instruments in centimeters, and an overall score automatically calculated by the simulator based on safety, economy of movement, and speed (scale: 0–20 points).

**Results:**

Operative time in an environment with classical music was significantly lower than in an environment with hard rock (95.9 s vs. 128.7 s, *p* = 0.0003). The overall rating in an environment with chatter was significantly lower than in a silent environment (11.7 vs. 15.7, *p* < 0.0001). The overall rating in an environment with hard rock was significantly lower than in an environment with classical music (14.3 vs. 17.5, *p* = 0.0008).

Surgeons who preferred listening to music in the operating room performed differently than those who did not. The mean operative time for surgeons who preferred music was 99.52 s (SD = 47.20), compared to 117.16 s (SD = 61.06) for those who did not prefer music, though this difference was not statistically significant (*p* = 0.082). The mean overall score for surgeons who preferred music was significantly higher at 17.46 (SD = 2.29) compared to 15.57 (SD = 3.49) for those who did not prefer music (*p* = 0.001).

**Conclusions:**

Our study suggests that exposure to classical music and silence may confer greater benefits to the surgeon compared to the impact of hard rock and chatter. These conclusions are grounded in significant differences observed in operative time and overall evaluations, highlighting the potential advantages of an environment characterized by acoustic tranquility for surgical professionals. Preferences for music in the operating room also play a role, with those who prefer music demonstrating better performance scores.

## Introduction

We investigated the impact of background auditory stimuli on the performance of surgeons training on an arthroscopy simulator, aiming to avoid any detrimental exposure for optimal learning.

Numerous studies have already explored the use of music in surgery, particularly its impact on surgeon stress and procedural efficiency. For example, few studies review the positive effects of music in reducing surgeon stress and improving procedural efficiency, indicating significant psychological and physiological benefits [1,2]. Additionally, a scoping review examines the effects of background music on the stress and anxiety levels of surgeons during operations, concluding that music generally has a positive impact [3].

Our study is distinct in specifically analyzing the effect of different types of background auditory stimuli on surgeons' performance during simulated arthroscopic tasks.

Simulators offer realistic and validated programs for surgical training, particularly for arthroscopy, enabling stress management and response to unexpected scenarios. The operating room, a critical environment for surgical practice, is characterized by the omnipresence of various auditory stimuli, including medical device alarms, conversations, and musical components. Despite recommendations from health authorities such as ARS (Agence Régionale de Santé) and WHO (World Health Organization) advocating for a noise level of 35 dB(A), the average noise in operating rooms typically ranges between 60 and 65 dB(A), occasionally exceeding 100 dB(A) [5,6]. Recognizing these disparities, it is widely accepted that maintaining a quiet sound environment in the operating room is essential to prevent negative impacts on all participants [[7], [8], [9]].

**Fig. 1:**
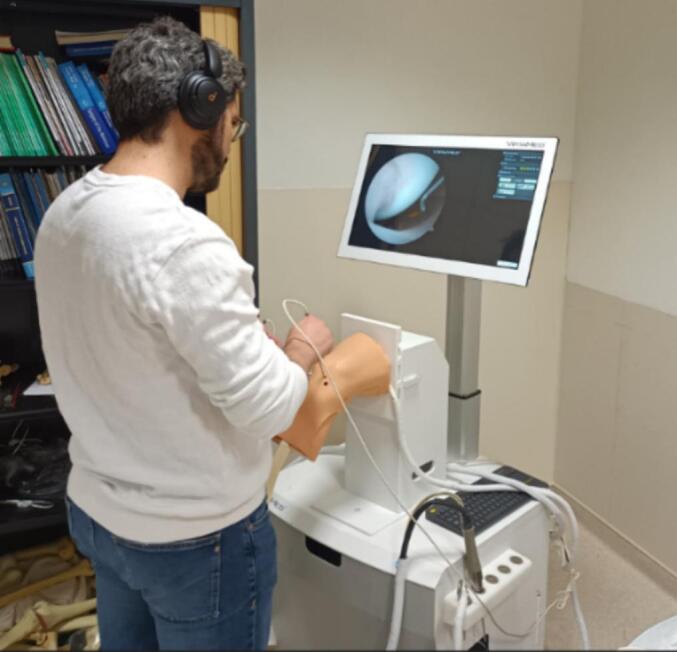
Example of participant using simulator.

This study employs the Virtamed ArthroS© simulator, specifically designed for knee, hip, ankle, and shoulder procedures. The primary objective of this investigation is to analyze the influence of the sound environment on arthroscopic surgical performance. Specifically, it aims to evaluate the effects of various types of background auditory stimuli on the performance of orthopedic surgeons during an arthroscopic simulation task (Fig. 1).

## Materials and methods

This prospective study conducted at a single center with a single observer spanned from November 2021 to November 2023, involving forty-seven orthopedic surgeons with varying arthroscopic expertise drawn from the population of arthroscopic surgeons in Haute-Normandie.

Participants with hearing impairments were excluded from the study.

The investigation focused on four distinct background auditory stimuli:

• **Quiet environment** achieved by setting the headphones (Soundcore Life Q30) to noise attenuation mode.
• **Classical music** characterized by Mozart's Piano Concerto No. 21.
• **Hard rock** using Airbourne's “Runnin’ Wild,” representing the rock music genre.
• **Ambient chatter** recorded from arthroscopist surgeons during breaks at the SFA Geneva 2021 congress spoken in French.

The study concentrated on four knee module exercises: “Triangulation skills III – rings,” “Catch the stars II,” “Guided meniscectomy I,” and “Loose body removal II.”

Data collection relied on the Virtamed ArthroS© simulator with background auditory stimuli transmitted through Soundcore Life Q30 headphones equipped with a noise attenuator set at 65 dB(A). The volume was controlled at the headphone output using a sound level meter (model XRCLIF), corresponding to a normal conversation threshold [10].

Each participant randomly and consecutively selected four modules and four auditory stimuli without redundancy, with one module and one auditory stimulus being paired each time (Fig. 4).

**Fig. 2:**
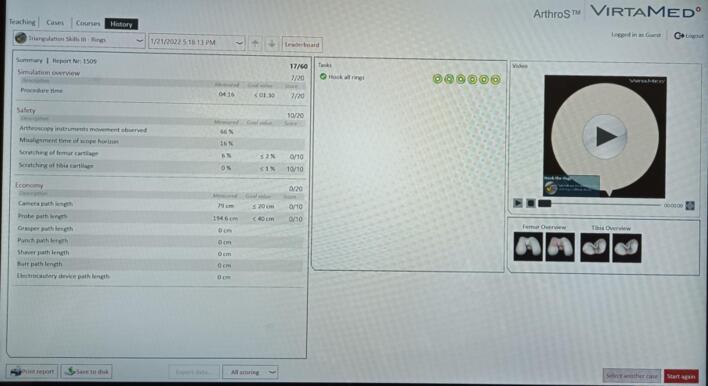
Example of data collected with simulator.

The investigation assessed various parameters including operating time in seconds and distances from the camera or instruments in centimeters. Following the simulator exercises, participants completed a musical questionnaire to provide additional insights into their experience. A table with the acoustic characteristics of each background auditory stimulus has been added to provide a better understanding of their temporal and frequency aspects. Psychoacoustic metrics such as loudness have also been included (Fig. 2).

**Fig. 3:**
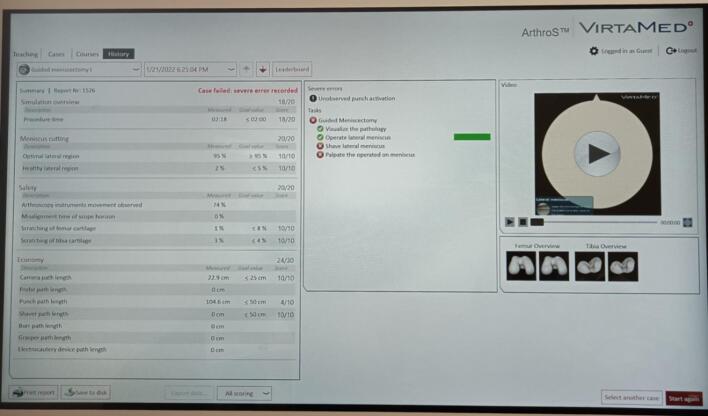
Simulator criteria analyses after training.

An overall score was also automatically calculated by the simulator for each exercise, based on criteria of safety, economy of movement, and speed, with a scale from 0 to 20 points (Fig. 3).

**Fig. 4:**
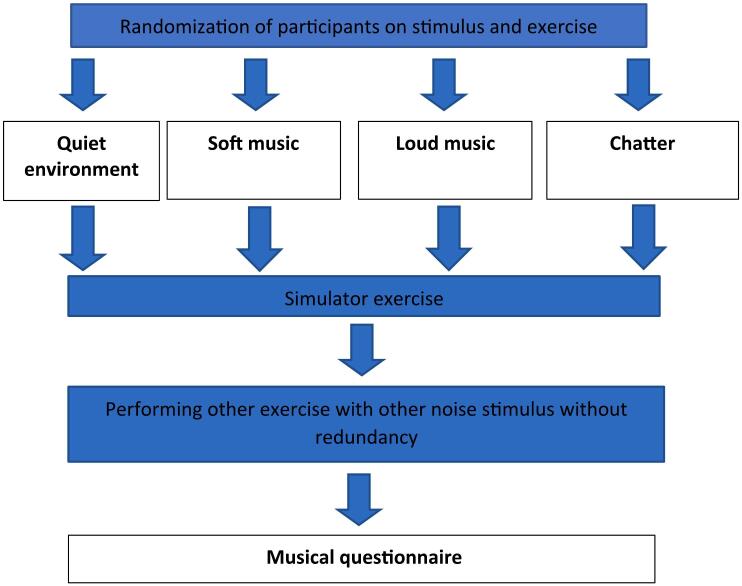
Study diagram.

A musical questionnaire was administered to specify the demographic and professional characteristics of participants and their musical habits (Table 1).

**Table 1:** Participant demographics with musical questionnaire.

Variables	Class	n (%)
Age	<30 years	35 (74.5)
	30–50 years	9 (19.1)
	>50 years	3 (6.4)
Sex	Male	39 (83.0)
	Female	8 (17.0)
Main dominant	Right handed	44 (93.6)
	Left handed	3 (6.4)
Professional status	Junior surgeon	35 (74.4)
	Clinical fellow	6 (12.8)
	Senior surgeon	6 (12.8)
Hospital	CHU of Rouen	41 (87.2)
	HPE - Le Havre	6 (12.8)
Level of experience in arthroscopy	Beginner	35 (74.5)
	Intermediate	7 (14.9)
	Advance	5 (10.6)
Do you listen to music during your free time?	Yes	44 (93.6)
	No	1 (2.1)
	Sometimes	2 (4.3)
Do you listen to music in the operating room?	Systematically	1 (2.1)
	No	2 (4.3)
	Sometimes	44 (93.6)
Would you prefer music played during surgery?	Yes	34 (72.3)
	No	13 (27.7)
What type of music do you prefer to listen to during surgery?	Classic	0 (0)
	Jazz	1 (2.1)
	Rock	10 (21.3)
	Electro	13 (27.7)
	Hip-Hop	0 (0)
	Variety	1 (2.1)
	Rap	19 (40.4)
	All types of music	1 (2.1)
	None	2 (4.3)
Is there a relationship between the type of music and the type of surgery?	Yes	1 (2.1)
	No	46 (97.9)
Should music be listened to systematically in the operating room?	Yes	5 (10.6)
	No	42 (89.4)
What volume music should be played in the operating room?	Low	20 (42.6)
	Medium	26 (55.3)
	Loud	1 (2.1)
Does the number of surgeries performed in the day affect your desire to listen to music?	Yes	3 (6.4)
	No	44 (93.6)
Does the time of the procedure affect your desire to listen to music?	Yes	4 (8.5)
	No	43 (91.5)
Does music affect communication in operating room?	Yes, better	2 (4.3)
	Yes, worst	29 (61.7)
	No effect	16 (34.0)
Does music in operating room make you:	Calmer	13 (27.7)
	More productive	17 (36.2)
	Disturbed	8 (17.0)
	Indifferent	9 (19.1)
Who chooses music in operating room?	Senior surgeon	45 (95.8)
	Junior surgeon	0 (0)
	Anesthetist	0 (0)
	Nurse	1 (2.1)
	Patient	1 (2.1)

Categorical data were reported as numbers and percentages. A paired Student's test was used to compare the average operating time and overall scores between two background auditory stimuli for the 47 surgeons included. Comparisons were made between silence and hard rock, silence and chatter, hard rock and classical music. *p* < 0.05 was considered as statistically significant.

Considering the multiplicity of tests, the significance threshold was lowered to 0.008 (alpha risk of 0.05 divided by the six comparisons performed). The statistical analysis was performed by the biostatistics team at the University Hospital of Rouen.

## Results

We included 47 surgeons. Demographic characteristics and musical habits are described in Table 1.

Among the study participants, 83 % were male and 17 % were female. The majority of respondents (74.5 %) were under 30 years old, 19.1 % were between 30 and 50 years old, and 6.4 % were over 50 years old. In terms of professional status, 74.4 % were junior surgeons, 12.8 % were clinical fellows, and 12.8 % were senior surgeons.

Most participants were from the Centre Hospitalier Universitaire de Rouen (87.2 %) and the Hôpital privé de l'Estuaire in Le Havre (12.8 %).

Regarding their musical habits, 93.6 % of participants regularly listen to music outside the hospital, and 93.6 % sometimes listen to music in the operating room (OR). Rock music was preferred by 21.3 % of respondents, followed by electronic music (27.7 %), rap (40.4 %), and jazz (2.1 %). No participant indicated a preference for classical music during surgery. Furthermore, 97.9 % of respondents reported no correlation between the type of music they prefer and the type of procedure performed.

When asked if music should be systematically played in the OR, 89.4 % of participants responded negatively. Most respondents (55.3 %) preferred listening to music at a medium volume, while 42.6 % preferred a low volume. The majority (61.7 %) stated that music worsens communication in the OR, while 34 % indicated it has no effect, and 4.3 % believed it improves communication.

Additionally, 36.2 % of participants reported that music made them more productive, 27.7 % felt calmer, 17 % felt disturbed by the music, and 19.1 % were indifferent. Regarding the choice of music, 95.8 % of participants indicated that the senior surgeon chooses the music in the OR.

### Results of operative time based on noise stimulus

The mean operative time and its standard deviation are summarized in Table 2.

**Table 2:** Exercises results according to sound stimulus.

Sound	Quiet environment	Classical music	Chatter	Hard rock
Participant (N)	47	47	47	47
Task time (s)	113.4 (59.3)	95.9 (47.7)	121.1 (63.1)	128.7 (66.4)
Camera path (cm)	48.3 (39.6)	39.0 (28.1)	52.1 (52.7)	46.7 (54.0)
Instrument distance (cm)	77.4 (42.0)	63.7 (29.7)	73.5 (31.9)	80.7 (47.7)
Overall test score	15.7 (4.3)	17.5 (2.2)	11.7 (6.7)	14.3 (6.2)

The comparison of mean operative time differences among various background auditory stimuli and the comparison of mean overall score differences among various background auditory stimuli are shown in Table 3.

**Table 3:** Statistical analysis of differences between sound stimuli.

	Task time			Overall test score		
Difference between 2 background auditory stimuli	Mean difference (s)	Confidence interval of difference	*P* value	Mean difference (s)	Confidence interval of difference	P value
Hard rock minus quiet environment	+15.29	[−5.29; 35.88]	0.1416	−1.42	[−3.59; 0.74]	0.1916
Chatter minus quiet environment	+7.74	[−13.53; 29.02]	0.4674	−3.98	[−5.68; −2.27]	<0.0001*
Hard rock minus classical music	+32.77	[15.85; 49.68]	0.0003*	−3.25	[−5.08; −1.42]	0.0008*

The asterisks denote statistically significant differences between the sound stimuli. Specifically: A P value marked with an asterisk (*) indicates a statistically significant difference, typically at the P < 0.05 level.

In this table: The comparison between “Chatter minus quiet environment” shows a P value of <0.0001*, indicating a statistically significant difference. The comparison between “Hard rock minus classical music” shows a P value of 0.0008*, also indicating a statistically significant difference. Therefore, the asterisks signify that the differences observed are statistically significant.

To determine if there is a significant difference in performance between individuals who prefer listening to music in the operating room (OR) and those who do not, we employed the independent two-sample *t*-test.

## Discussion

The present study has some weaknesses, including a population consisting of young male surgical students and the absence of a control group, which may limit the generalizability of the conclusions to a broader population. However, the study's strength lies in its methodological homogeneity, prospective single-center design, and meticulous adherence to a standardized protocol. It provides valuable insights into the impact of background auditory stimuli on surgical performance. The research was conducted using a unified simulator and module, with a homogeneous cohort of right-handed male junior surgeons predominantly inexperienced in arthroscopy.

Notably, the participants exhibited a preference for music in their personal lives, with a prevailing inclination towards rap, rock, and electro genres. The majority advocated for a moderate intensity of music during surgical procedures, although unanimity prevailed against the systematic incorporation of music into the operating room ambiance.

Our main hypothesis was that background auditory stimuli had a direct impact on the surgeon regardless of his level of experience, positive through the Mozart effect or negative through chatter and hard rock. The Mozart effect is based on the hypothesis that listening to music could induce a short-term improvement in certain types of mental tasks called “spatio-temporal reasoning” [11].

Analysis of operative times revealed a distinctive hierarchy: the most favorable times were achieved in a classical music environment, followed by a silent environment, chatter, and conversely, the longest durations during exposure to hard rock. This pattern aligns with existing literature substantiating the influence of background auditory stimuli on surgical efficiency. Variations in camera and instrument distances were observed across different background auditory stimuli. Akin to operative time trends, a hierarchy emerged: quiet environments and classical music exhibited more favorable distances, while chatter and hard rock were associated with less optimal spatial parameters.

Simulator-derived scores reflected a parallel pattern. Classical music yielded the highest mean score, followed by silence, hard rock, and the lowest score associated with chatter. This underscores the potential impact of background auditory stimuli on the quality of technical acts performed during arthroscopy. Despite the surgeon's subjective receptivity to music, the findings illuminate the potential detrimental effects of noise stimuli on arthroscopic tasks. Chatter, in particular, emerged as a significant source of distraction and risk, evidenced by ten participants who failed tasks due to safety faults resulting in immediate zero scores.

Furthermore, the analysis showed that surgeons who preferred listening to music in the OR had better performance scores. Although the difference in operative times between those who preferred music and those who did not was not statistically significant, the overall scores were significantly higher for those who preferred music. This suggests that personal preferences for music in the OR can influence performance positively (Table 4).

**Table 4:** Comparison of the impact of listening to music on operative time and global score, considering participants music habits.

	With music (Mean ± SD)	Without music (Mean ± SD)	Mean difference	Confidence interval	Test statistic	*p*-Value
Operative time	99.52 ± 47.20	117.16 ± 61.06	−17.64	[−5.29; 35.88]	−1.751	0.082
Global score	17.46 ± 2.29	15.57 ± 3.49	1.89	[−3.59; 0.74]	3.357	0.001

Existing studies, including those by Weldon et al. [14] and Kurmann [9], corroborate the multifaceted influence of noise stimuli on surgical performance. Weldon's quantitative analysis demonstrated increased operating time and tension with music, while Kurmann's work emphasized a potential link between stimuli and surgical site infections (SSI). Lies and Zhang [12] also demonstrated the beneficial effect of music on surgeon performance by reducing task time by 10 % for junior surgeons and 8 % for senior surgeons. Wiseman [13] demonstrated the same improvement in coordination and performance of technical acts during arthroscopy with the discovery of a Mozart effect present in two forms: quantitative with the reduction in operating time and qualitative with better performance.

The WHO [6] recommended a sound level not exceeding 30 dB(A) aligns with the observed adverse effects of higher noise levels on patient safety. The Mozart effect, hypothesizing a short-term improvement in spatio-temporal reasoning through music, found support in our findings. Classical music played at a moderate level demonstrated potential benefits without impeding communication skills. Conversely, Trappe [15] suggested that genres such as rock, heavy metal, and techno were associated with increased heart rates in surgeons and activation of the noradrenergic system, potentially contributing to stress and arrhythmia. Notably, most exercise failures in our series were linked to rock stimuli or chatter, substantiating the potentially disruptive impact of specific noise stimuli.

## Conclusion

Our study underscores the significant impact of background auditory stimuli on surgical simulator performance. Preferences for music genres, adverse effects of chatter on safety, and the relevance of the Mozart effect were observed. The hierarchy in operative times and simulator scores emphasizes the need for a tailored noise environment for learning surgeons. These insights advocate for individualized interventions and align with global health recommendations emphasizing the crucial role of noise practices in optimizing surgical outcomes. Future studies could enhance the precision of surgical procedures and contribute to the overall well-being and safety of both surgical teams and patients.

## Funding sources

No funding sources were used to write this work.

## Ethics approval

All the surgeons have given their consent for this scientific study.

## CRediT authorship contribution statement

**Alexandre Czerwiec:** Writing – review & editing, Writing – original draft, Visualization, Validation, Methodology, Investigation, Funding acquisition, Formal analysis, Data curation, Conceptualization. **Margot Vannier:** Writing – review & editing, Methodology. **Olivier Courage:** Validation, Supervision.

## Declaration of competing interest

None of the authors have conflicts of interest related to this work.
